# Design and Preliminary Findings From a New Electronic Cohort Embedded in the Framingham Heart Study

**DOI:** 10.2196/12143

**Published:** 2019-03-01

**Authors:** David D McManus, Ludovic Trinquart, Emelia J Benjamin, Emily S Manders, Kelsey Fusco, Lindsey S Jung, Nicole L Spartano, Vik Kheterpal, Christopher Nowak, Mayank Sardana, Joanne M Murabito

**Affiliations:** 1 Cardiology Division, Department of Medicine University of Massachusetts Medical School Worcester, MA United States; 2 Department of Quantitative Health Sciences University of Massachusetts Medical School Worcester, MA United States; 3 Department of Medicine University of Massachusetts Medical School Worcester, MA United States; 4 Boston University's and National Heart, Lung, and Blood Institute's Framingham Heart Study Framingham, MA United States; 5 Department of Biostatistics Boston University School of Public Health Boston, MA United States; 6 Section of Preventive Medicine and Epidemiology and Section of Cardiovascular Medicine Department of Medicine Boston University School of Medicine Boston, MA United States; 7 Department of Epidemiology Boston University School of Public Health Boston, MA United States; 8 Section of Endocrinology, Diabetes, Nutrition, and Weight Management Boston University School of Medicine Boston, MA United States; 9 Care Evolution Ann Arbor, MI United States; 10 Cardiology Division Department of Medicine University of California San Francisco San Francisco, CA United States; 11 Section of General Internal Medicine, Department of Medicine Boston University School of Medicine Boston, MA United States

**Keywords:** smartphone, tele-medicine, blood pressure monitoring, ambulatory, cohort studies

## Abstract

**Background:**

New models of scalable population-based data collection that integrate digital and mobile health (mHealth) data are necessary.

**Objective:**

The aim of this study was to describe a cardiovascular digital and mHealth electronic cohort (e-cohort) embedded in a traditional longitudinal cohort study, the Framingham Heart Study (FHS).

**Methods:**

We invited eligible and consenting FHS Generation 3 and Omni participants to download the electronic Framingham Heart Study (eFHS) app onto their mobile phones and co-deployed a digital blood pressure (BP) cuff. Thereafter, participants were also offered a smartwatch (Apple Watch). Participants are invited to complete surveys through the eFHS app, to perform weekly BP measurements, and to wear the smartwatch daily.

**Results:**

Up to July 2017, we enrolled 790 eFHS participants, representing 76% (790/1044) of potentially eligible FHS participants. eFHS participants were, on average, 53±8 years of age and 57% were women. A total of 85% (675/790) of eFHS participants completed all of the baseline survey and 59% (470/790) completed the 3-month survey. A total of 42% (241/573) and 76% (306/405) of eFHS participants adhered to weekly digital BP and heart rate (HR) uploads, respectively, over 12 weeks.

**Conclusions:**

We have designed an e-cohort focused on identifying novel cardiovascular disease risk factors using a new smartphone app, a digital BP cuff, and a smartwatch. Despite minimal training and support, preliminary findings over a 3-month follow-up period show that uptake is high and adherence to periodic app-based surveys, weekly digital BP assessments, and smartwatch HR measures is acceptable.

## Introduction

### Background

Despite its great contributions to our understanding of cardiovascular disease (CVD) and its risk factors, traditional cohort epidemiology can be costly and time consuming [1-3], and it has been recently criticized for a lack of integration of “ *real world* health information, such as activity level, heart rate (HR) , or home blood pressure (BP) [4]. Recommendations for launching digital epidemiology have been put forth by a working group from the National Heart, Lung, and Blood Institute on Epidemiology and Population Science with the need for evaluation of scalability and reliability of digital and mobile health (mHealth) data [5]. The rapid evolution of commercial mHealth technologies presents a transformational opportunity for CVD phenotyping outside of the clinical and Research Center settings [6-8]. Nevertheless, to date, electronic cohorts (e-cohorts) have largely enrolled middle-aged and younger volunteers with a low prevalence of CVD and its risk factors [9]. With the National Institutes of Health’s *All of Us* Program, plans to engage substantial numbers of participants electronically are evolving [10], but, to date, a few ongoing studies have successfully enrolled participants in mHealth research [9,11]. Furthermore, little is known about how mHealth and digital CVD phenotypes, such as activity level, relate to other CVD risk factors and outcomes in middle-aged and older adults [12].

### Objectives

**Figure 1:**
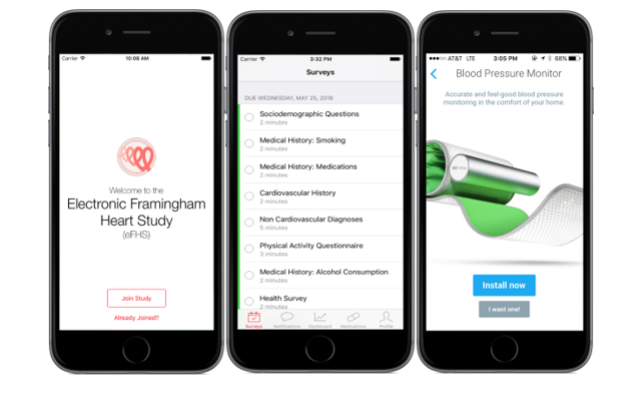
Screenshots of the electronic Framingham Heart Study app loading screen, questionnaire, and interface with digital health device (blood pressure monitor).

In this paper, we describe the design of the electronic Framingham Heart Study (eFHS). The broad objective of the eFHS is to add new mobile and digital phenotypes into the rich Framingham Heart Study (FHS) and leverage its deep CVD risk factor phenotyping, longitudinal follow-up, and established relationships with its participants. We plan to compare novel measures obtained from commonly used commercial mHealth and digital devices with other gold standard CVD phenotypes obtained in the FHS Research Center as well as CVD outcomes. We have developed a custom smartphone app, the *eFHS* app, (see Figure 1), that is capable of messaging participants, administering surveys to collect health history and behavior updates, and pulling data from co-deployed wearables, including a digital BP cuff and smartwatch. The research center staff are deploying the eFHS app and devices and training participants on system components in the context of their scheduled and ongoing FHS Research Center examinations. In addition to describing our approach and methodology, we report the characteristics of the eFHS participants, rates of adherence to the eFHS survey, and digital BP and smartwatch research protocols over the 3 months since deployment.

## Methods

### Study Cohort

The eFHS is enrolling participants from the Framingham Heart Study (FHS) Third Generation Cohort (Gen 3, total n=4095), multiethnic Omni Group 2 Cohort (Omni 2, total n=410), and New Offspring Spouse Cohort (NOS, n=103) enrolled from 2002 to 2005, who are examined every 6 to 8 years, with details previously reported [13]. eFHS is leveraging the in-person NOS Examination 3 in the Omni 2 in the FHS Gen 3 that began in April 2016 in the FHS Research Center [14]. eFHS began enrollment on June 20, 2016, with the smartphone app and a digital BP cuff for weekly home BP monitoring. A smartwatch for daily activity and HR monitoring was added on November 17, 2016. The eligible participants were invited to participate in eFHS as 1 component of the routine research center examination, and they were allowed to decline the use of both digital devices. To be eligible for eFHS, English speaking participants were required to own an iPhone with a compatible iOS (version 9 or higher), reside in the United States, and be willing to provide permissions for notifications and data sharing with the Research Center. All participants provided informed consent to participate in the eFHS, both as part of the overall consent for Examination 3 and an electronic consent within the eFHS app itself (2-level consent, the consent forms are available on the FHS website [15,16]. The eFHS protocol was reviewed and approved by the FHS Executive Committee and the Institutional Review Board at the Boston University Medical Center.

**Figure 2:**
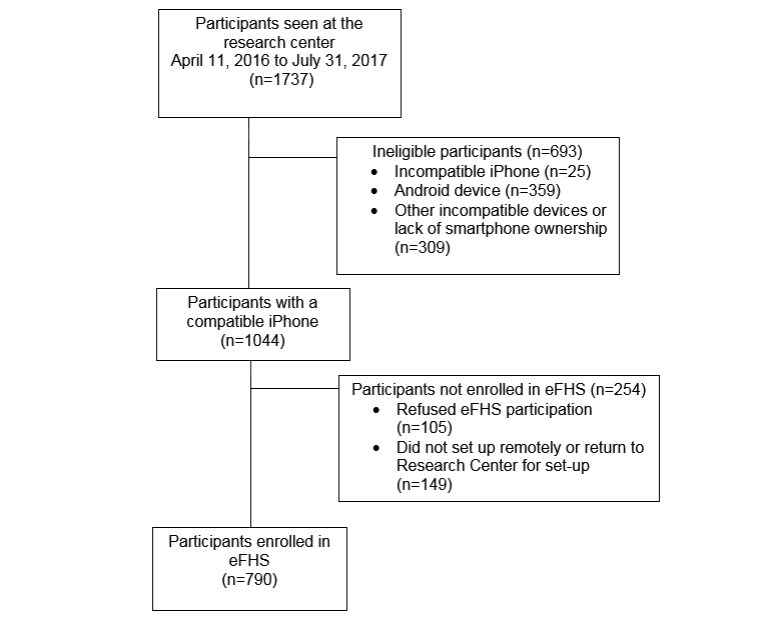
Flow diagram depicting the enrollment of participants in electronic Framingham Heart Study and reasons for nonenrollment.

As of June 2018, enrollment in eFHS is still ongoing, it will continue until all FHS participants attend Examination 3. From June 2016 to July 31, 2017, we have enrolled 790 participants in eFHS. There were 1737 FHS participants examined at the Research Center. Of these, 1044 FHS participants (60%) owned a compatible iPhone and were considered eligible for enrollment in the eFHS (Figure 2). Of those who were ineligible, 359 (21%) were Android operating system (OS) smartphone owners and 25 (1%) used an iPhone running an iOS version too old to be compatible with the eFHS app. In addition, 105 eligible FHS participants (10%) declined participation in eFHS and 149 who initially agreed did not complete the setup of the app. Of the enrolled participants, 689 (64%) downloaded the app and paired the devices in the Research Center with study staff on the day of their scheduled study examination. A total of 21 eFHS participants out of the 790 (2%) returned after their examination for a dedicated eFHS training session and device set up in the Research Center, and the remainder were provided with study devices and instructions by study staff and they set up remotely.

### Study Smartphone Application

We developed a custom smartphone app (Figure 1,Multimedia Appendix 1) by using Apple’s ResearchKit with the assistance of an industry partner (CareEvolution). The app was designed to electronically distribute and collect survey data and communicate with participants outside of the FHS Research Center. The app can be downloaded from the App Store. The study staff assist participants to download the eFHS app, either via written instruction or via direct assistance in the Research Center. The first-time the participants downloads the app, they are prompted to log in and register as an eFHS participant. They are then asked to provide an electronic informed consent and provide notification permissions. Health surveys are administered through the app at baseline and then at 3-month intervals (Multimedia Appendix 2). The eFHS app also prompts health access permissions that enable pairing with mobile and digital health devices, including the smartwatch and BP cuff distributed to participants. All participants receive a welcome to the eFHS message, reminders when surveys are due, notification of new activities, and reminder messages to comply with study protocols, including weekly home BP measurements and daily smartwatch use, as appropriate.

Once participants download the eFHS app, they are prompted to complete surveys that include digital versions of several standardized assessments performed in the Research Center. There are 9 baseline surveys (Multimedia Appendix 2) that request demographic information, as well as information about level of physical activity (FHS physical activity index), health-related behaviors (ie, alcohol consumption, smoking), medication use, mood (Center for Epidemiologic Studies Depression Scale, CES-D) [17], CVD risk factors, and baseline CVD and other health conditions. Additional abbreviated surveys are being distributed at 3, 6, 9, and 12 months after enrollment. At 3 months, 1 survey is deployed to assess the level of self-reported physical activity. Additional information, including a health history update, mood and function assessments (CES-D), health survey, and a repeat assessment of physical activity and mobility are planned for the 6-month digital surveys (Multimedia Appendix 2).

### Study Digital Devices

In addition to the eFHS app, eFHS participants are offered 2 digital devices as part of the eFHS: (1) the Nokia-Withings digital BP cuff model BP-801 (beginning June 2016, Figure 1) and (2) the Apple Watch Generation 0 (beginning November 2016). The Nokia-Withings BP device was selected because it is Food and Drug Administration–approved for home BP monitoring, has been validated (accuracy ±3 mmHg) [18], and has been used in other digital device studies [9]. The Apple Watch was selected because of its compatibility with the Apple iOS, because it is one of the most commonly used wrist activity monitors capable of both activity and HR monitoring and because it has been shown to be the most accurate commercial wrist-worn HR and a highly accurate step monitor [19].

All eFHS participants are instructed on the proper use of the digital BP cuff and instructed to perform weekly home BP measurements. When feasible, the research staff ask participants to perform their first digital BP in the Center to ensure understanding of the BP protocol and functions of the digital BP cuff. Participants are provided with written instructions on the use of the digital cuff as well as proper home BP monitoring techniques [20]. Participants are asked to take their digital BP once per week on the same day at about the same time, if possible. Participants are provided with written BP guidelines for use to contact their health care provider if readings are elevated. In 2016, during the study period, Withings was purchased by Nokia. A new smartphone app (Nokia Health Mate) was required to sync the digital BP cuff with the eFHS app. The research staff contacted participants and helped them download the new app to ensure continued adherence with study protocols. Additional BP recordings obtained by participants are also collected and transmitted to the Research Center.

All eFHS participants are instructed on the proper use of the Apple Watch. When possible, the research staff synchronize the participant’s smartphone with the eFHS app and Apple Watch in the research center. When this is not possible, the participant synchronizes the device at home. Beginning in 2017, the Apple Watch Generation 0 required an update be installed on it before it could be paired with any iPhone. Initially, participants were asked to take the smartwatch home after this change occurred because of time constraints and were provided with written instructions. The study staff then obtained dedicated study iPhones to download this Apple Watch upgrade onto all study smartwatches before they were deployed. As part of the eFHS research protocol, participants are asked to wear the smartwatch daily and are sent home with instructions on proper smartwatch use and charging.

### Study Reminders

Participants receive scripted reminders (Multimedia Appendix 3) through the app at critical points. For example, when joining the eFHS, a notification message is sent as follows: “Welcome to the Electronic Framingham Heart Study. During the next two weeks, please complete your initial surveys available on the surveys tab below.” Participants also receive messages when new surveys become available, “There are new surveys available to complete. Please open the eFHS app and complete them.” Reminder notifications are sent when surveys come due, for example, “Reminder: You have surveys to complete” or “You have surveys due today. Please open the eFHS app and complete them.” For participants who had some incomplete surveys and then completed all surveys the following message is sent, “Thank you for completing all your surveys. Your contribution is a vital part of our ongoing research efforts! Emily and the FHS team.”

### Data Management

**Figure 3:**
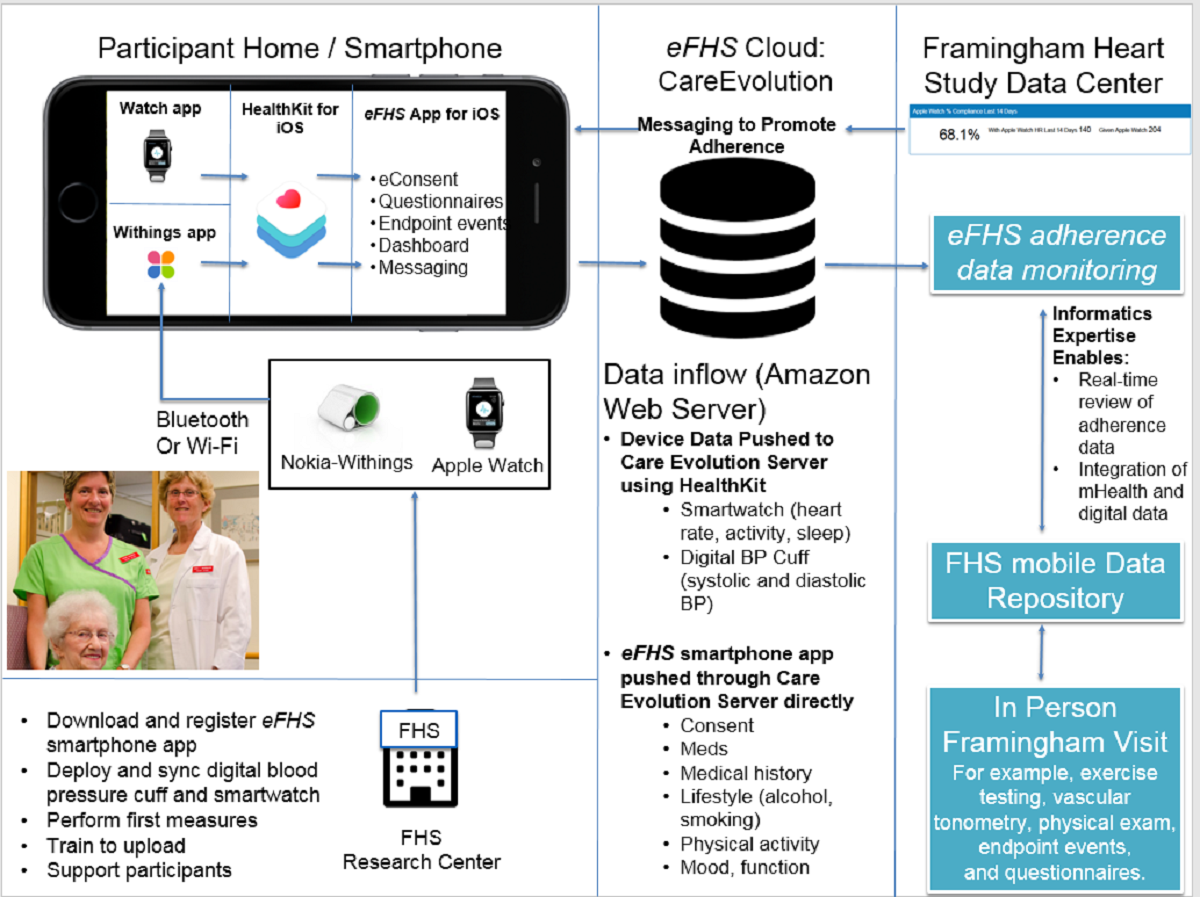
Electronic Framingham Heart Study (eFHS) components (eFHS smartphone app, digital blood pressure cuff, and smartwatch) and data infrastructure. BP: blood pressure; eFHS: electronic Framingham Heart Study.

All data acquired as part of the eFHS are stored on the participant’s mobile device until the participant has a stable data connection. Then, study data are passively pushed to a secure cloud server in the United States in a Health Insurance Portability and Accountability Act compliant fashion (Figure 3). Adherence to study protocols is monitored by the study team. Data labeled with the participant’s eFHS ID are batched and transmitted to the FHS Research Center servers. FHS statisticians perform quality control activities and further prepare data for analyses.

### Study Analyses

Primary eFHS outcome variables include the following: survey, BP, wearable device, and overall adherence over 12 months. In prespecified analyses, we will identify predictors of successful eFHS protocol adherence to inform future mHealth research. We also plan to explore relations among validated measures of cardiovascular health obtained in the FHS Research Center during examination 3, including measures of vascular stiffness and exercise capacity from formal cardiopulmonary exercise testing, with home BP, and average daily HR and steps, respectively.

For the present descriptive analysis, we described the characteristics of all eFHS participants enrolled as of July 31, 2017 and compared them with exam 3 participants who did not participate (either ineligible or declined). Baseline characteristics of all eFHS and other exam 3 participants are presented as means ± standard deviation for continuous variables and as numbers and percentages for nominal variables.

We examined completion rates of the baseline and 3-month surveys and use of digital devices at baseline and 3 months. We restricted our analysis of survey completion to eligible participants (baseline, n=790) and 3-month surveys (n=790). We report the proportion with successfully uploaded survey data (some survey completed, entire survey completed, and no survey completion) for the digital baseline survey (comprising 9 questionnaires, Multimedia Appendix 2) and survey completion at 3 months (comprising 1 questionnaire, Multimedia Appendix 2). Similarly, we examined adherence to weekly home BP monitoring only among participants who consented to take the Nokia Withings BP cuff (n=573) as part of the eFHS. Similarly, we assessed smartwatch use only among participants who took an Apple Watch and consented to wear it daily as part of the eFHS (n=405). We examined the weekly proportion of participants with at least one BP and HR recording, respectively.

## Results

### Characteristics of Electronic Framingham Heart Study Participants

Between June 2016 and July 2017, we enrolled 790 participants in eFHS. Due primarily to a lack of compatible smartphone, 947 additional FHS participants were not enrolled (Figure 2). eFHS participants were similar to FHS participants who were ineligible (ie, incompatible smartphone) or declined to participate in the eFHS with respect to their race. However, eFHS participants were slightly younger, more likely to be female, attained a higher level of education, more likely to report being in excellent health, and were less likely to be affected by CVD or its risk factors than those who were ineligible or did not participate (Multimedia Appendix 4).

**Figure 4:**
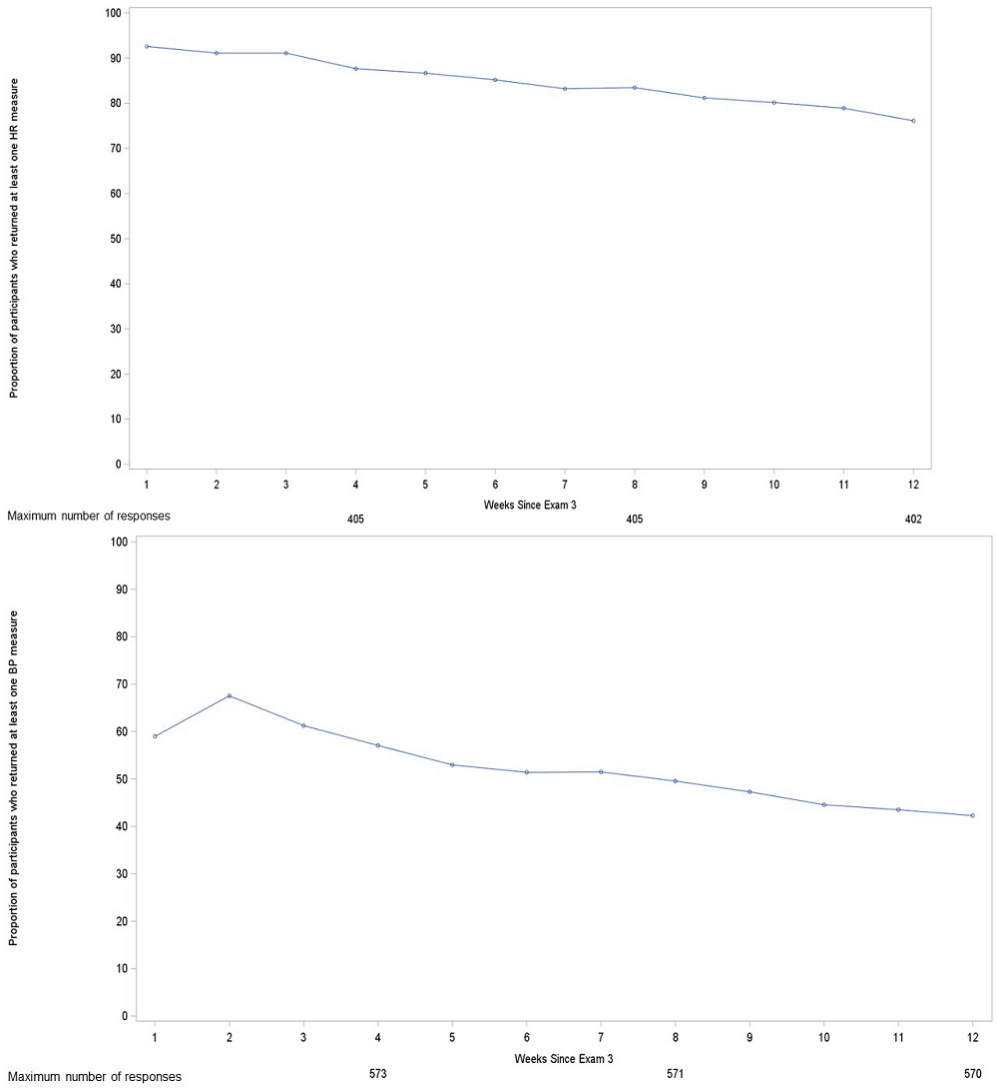
Proportion of eFHS participants uploading at least A) one digital BP weekly or B) one heart rate weekly over the 12 weeks following enrollment.

### App and Device Deployment and Adherence

As shown in Multimedia Appendix 5, out of the 790 (85%) eFHS participants completed all the baseline surveys and an additional 38 out of the 790 participants (5%) completed some of the baseline surveys. A total of 470 out of the 790 (59%) eligible participants completed the 3-month survey. The average time to complete all of the baseline and 3-month digital surveys was modest (18 min for the baseline surveys and 2 min for the 3-month survey). Relating to the staggered rollout of study devices and delayed smartwatch availability, a greater number of eFHS participants received a digital BP cuff (n=573) than those who received a study Apple Watch (n=405). We did not collect data on the reasons why some participants opted to take 1, both, or no smart devices, because of logistical constraints. However, based on the communications with the FHS Research Center staff, we believe that choices were partly influenced by the perceived time burden imposed by home BP or wearable device use.

Some digital BP or HR data were obtained from 79% (n=626) of the 790 eFHS participants who elected to take at least one study device. Just under half (44%) of the participants (n=156/353, received the BP cuff, smartwatch and app) transmitted both BP and HR recordings during the 12th study week. Adherence to the use of the study smartwatch (Figure 4, as defined for study purposes as uploading at least one HR value during the week, was appreciably (about 30% higher) and consistently higher over the 3 months following enrollment than the adherence to the use of the digital BP cuff (Figure 4), as defined as uploading at least one BP value during the week. We observed slightly higher rates of adherence to BP monitoring but no significant difference with respect to rates of adherence to HR monitoring among eFHS participants with a previous history of hypertension or cardiovascular disease as compared with those free from hypertension or CVD in our sample (Multimedia Appendix 6).

## Discussion

### Study Background and Design

The eFHS is a novel e-cohort embedded in the ongoing prospective FHS of middle-aged Gen 3 and Omni-group 2 cohort participants attending an in-person study examination. We have described the methodology for recruitment of the eFHS cohort, the characteristics of this cohort after 1 year of enrollment, as well as survey completion and adherence to a digital BP cuff and a smartwatch (for once weekly home BP and HR measurement, respectively) over 3 months of follow-up.

Our decision to target the FHS Gen 3 and Omni 2 cohorts for participation in the creation of an e-cohort was substantially informed by 2 previous studies. First, a digital connectedness survey was conducted in 2014 to quantify internet access and device use among FHS participants, including computers, internet, smartphones, wireless devices, and apps [14]. We received information from 6503 FHS participants: a total of 87% (n=5678) of our participants had internet access and 60% (n=3907) used smartphones. We observed that 69% (n=2689) of smartphone users owned iOS (Apple) compatible devices, 22% (n=855) had Androids, and most smartphone users were FHS Gen 3 participants [14]. Second, in 2016, we conducted a randomized controlled trial (NCT02531763) among FHS families using a smartphone app (Moves or Fitbit) and a wrist-based activity monitor (Fitbit) in the *B* ehavioral *E* conomics *F* ramingham *I* ncentive *T* rial, *BE FIT*, study [21]. FHS participants randomly assigned to the intervention arm entered a 12-week game to achieve physical activity step goals. Among the 200 trial participants, 97% completed the study. The BE FIT study enabled us to (1) gain experience deploying a smartphone app and wearable devices, (2) engage FHS participants in mHealth activities (89% of the intervention arm and 83% of the control arm agreed or strongly agreed to the statement “I enjoyed participating”), and (3) determine that participants were highly likely to adhere to device use over at least 3 months.

The enrollment and retention rates in epidemiology studies have declined over the last 2 decades [22,23]. Furthermore, the financial and logistical burdens associated with traditional cohort-based epidemiology are substantial [24]. Finally, cohort participants spend just 4 hours in the research center every 6 to 8 years or a few hours per year with their designed health care provider, but they spend 5000 waking hours each year making decisions that can profoundly affect health (eg, to stop smoking, to engage in regular physical activity, and to take prescribed medications to treat BP) [25]. Real-world data obtained from participants over extended periods can potentially enhance cardiovascular risk factor identification and prediction of incident CVD [10]. To that end, development of electronic epidemiological cohorts, or e-cohorts, whereby information is collected electronically by leveraging Web-based platforms, smart devices, or smartphone-compatible applications, offer potentially sustainable alternatives in this era of digitization [9,26-29].

In contrast to several other e-cohorts that primarily use Web-based platforms for enrollment and interaction [9,26-29], we elected to develop a novel app for survey administration and deploy it for use in the context of a scheduled study exam conducted at the FHS research center. Electronic surveys administered using Web-based platforms are fast to administer and easier to code than smartphone apps [30,31]. In a recent analysis conducted by the Pew Research Center, general response rates for study participants using smartphone app-based surveys were lower than the Web-based surveys (any survey completed by 76% vs 94%, in app vs Web-based group, respectively; all surveys completed by 12% vs 17%, in app vs Web-based group, respectively) [32]. However, smartphone app-based surveys offer several advantages, including ease of administration, integration of data from other smart devices, and collection of *real-time* responses that may occur outside the home or office and thereby suffer from less recall bias than Web-based surveys [32].

### Study Enrollment

Just under half (n=790, 48%) of the 1737 FHS participants enrolled in the eFHS, a rate lower than has been observed in other e-cohorts (ie, Health eHeart, 78% of registered participants completed the consent, 86% of consented completed at least one survey, 37% of consented participants completed all surveys) [9]. Nevertheless, only 105 participants (10%) of the 1044 FHS participants with a compatible smartphone declined eFHS participation and the vast majority of FHS Gen 3 and Omni 2 participants who were eligible agreed to enroll, with ownership of an Android OS smartphone being the major reason for not being enrolled (Figure 2). To address this problem, we have recently developed an eFHS app for the Android OS and are now actively enrolling these participants into the eFHS. Furthermore, once enrolled and consented, survey completion rates are higher than some *de novo* e-cohorts [33]. We hypothesize that embedding enrollment in the Research Center of an ongoing cohort was instrumental in promoting initial enrollment and facilitating high rates of survey completion through in-person training.

### Study Adherence

Among the 790 eFHS participants, we observed that the high rates of completion for our eFHS app-administered baseline survey (90%; 713 participants completed at least some of the baseline survey) and 3-month survey were 59% (n=470), with little reinforcement. The high completion rate is notable in light of the feature that participants needed to keep the eFHS app open to receive any eFHS notifications, including a reminder to complete the surveys. The high rates of survey completion likely resulted from app download in the research center for most participants as well as through optimization of our app survey methodology to avoid respondent fatigue. We optimized the digital surveys through the use of progress indicators, by limiting the number of answer options and allowing participants to complete the questionnaire across multiple sittings and by restricting the number of questions per survey to 10 to 20 [34]. Compared with other contemporary e-cohorts with ongoing data collection, our rates of survey completion were higher than that those in previous studies, including eCARDIA (52% survey completion) [29] and the Health eHeart Study (37% completed all surveys and 29% of myHeart participants completed all surveys) [9]. Our findings suggest that linking e-cohort enrollment to a traditional research center study visit offers distinct advantages with regard to higher rates of adherence to digital health survey completion. We are developing interactive messaging functionalities as part of an eFHS dashboard in an effort to boost survey completion and promote long-term adherence to survey completion.

### Digital Device Deployment and Adherence

The majority, 573 (73%), of the 790 eFHS participants agreed to use a digital BP cuff weekly and over half, 405 out of the 790 participants (51%), agreed to wear a smartwatch daily. The difference in device acceptance is related to the delayed start for the smartwatch. Once the smartwatch deployment began, acceptance of the 2 devices was similar. Adherence to device use was initially high, likely secondary to the significant technical support provided to eFHS participants, particularly those who had their digital devices set up and synchronized with the eFHS app in the FHS research center. However, digital BP use waned significantly over the 3 months following enrollment, with fewer than half of participants uploading a BP value in the 12th study week. Digital BP adherence rates in our study were slightly lower than those seen in another recent study, in which the rate of adherence to twice-monthly home BP monitoring (iVitality) was 64% over a 6-month period [35]. We required participants to upload a BP more frequently and our eFHS sample of 790 participants had a relatively low prevalence of diagnosed hypertension (24%, n=186), which may have contributed to the lower rate of study protocol adherence as compared with this smaller e-cohort study involving participants enrolled via the Web. This hypothesis appears to be supported by the slightly higher rate of adherence among participants with a history of hypertension in our cohort (Multimedia Appendix 5). However, our findings should be interpreted with caution in light of the limited number of participants with a history of CVD in our sample.

The rates of smartwatch use were significantly higher than the rates of digital BP use throughout the first 12 weeks of the study despite the release of an iOS update during this period that interfered with uploading HR values from some Apple Watches. Differences in rates of adherence to the digital BP cuff and smartwatch are likely attributable to the passive nature of data acquisition from the smartwatch and the value to participants from having a functional watch [36].

### Study Challenges

Our team encountered several technical challenges as the eFHS progressed. Participants *forced closed* the eFHS app unknowingly, preventing transmission of BP and HR data. We updated our in-person training and written materials to advise participants to keep the eFHS app open. The BP cuff manufacturer Withings changed to Nokia and we encountered a brief period of difficulty downloading and configuring the BP app with the device. A new version of the Nokia Health Mate app was released, and that resolved the problem we encountered. Finally, there was an iOS update to the Apple Watch that significantly added to the time to pair the watch and iPhone in the Research Center with staff support, leading many participants to opt to take the device and instructions home to set up independently. The Research Center staff have obtained dedicated study iPhones to update the watches before the in-person examinations to speed the synchronization.

### Differences between Electronic Framingham Heart Study and Framingham Heart Study Participants

In a manner consistent with other digital or e-cohorts, as compared with other Gen 3 and Omni 2 FHS participants who were ineligible or declined participation, eFHS participants were younger, more likely to be women, and had more favorable socioeconomic determinants of health and better self-reported overall health status and lower prevalence of cardiovascular risk factors [9,11,26-28]. Our findings mirror Pew data on iOS device ownership and have implications for recruitment of participants for the National Institutes of Health’s *AllofUS* initiative, as methods to address sampling biases might be needed to obtain representative data from e-cohorts that are applicable to the overall US population [10]. We are attempting to create a more generalizable e-cohort by developing enhancements to our app, and we will reexamine differences among eFHS versus other FHS participants who are ineligible or decline participation after we complete the enrollment of participants with Android OS–based devices.

### Future Directions

The FHS represents a useful setting to define adherence to novel mobile methods for cardiovascular phenotyping using mobile and digital devices among older adults at risk for CVD. Once device deployment and follow-up are complete, we plan to analyze long-term rates of survey, BP, and wearable device adherence over 12 months. In subsamples of eFHS participants, we intend to study the effectiveness of various mHealth supportive interventions, such as personalized short message service text messaging, on protocol adherence. Finally, we plan to leverage the richness of the FHS through in-person examination completed by all study participants on the day of their enrollment. We will analyze relations among validated measures of cardiovascular health, particularly markers of vascular stiffness and exercise capacity, home BP, as well as HR and activity. The overarching goal of the eFHS will be to inform clinical practice and research of how mHealth and digital health measures relate to cardiovascular health and CVD risk.

### Study Strengths and Limitations

Our study findings should be considered in the context of their strengths and limitations. We are developing an eFHS cohort from the Gen 3 and Omni 2 (multiethnic) participants who are presenting to the research center for an in-person scheduled examination. In so doing, we have enhanced our ability to enroll and train participants on app and device use, as well as to correlate findings from digital devices with the data collected during the in-person examinations. Furthermore, we were able to attain high rates of digital survey completion using a novel smartphone app and acceptable rates of home device use over 3 months, all with limited post enrollment technical support and minimal use of push notifications or reminders. However, our study has certain limitations. First, the eFHS cohort largely comprises middle-aged, white participants—mean age 53 (8) years—and, enrollment rates, survey completion rates, and digital device home use adherence rates observed in our study might not be generalizable to the samples with older cohort participants or cohorts involving a larger proportion of individuals of other racial/ethnic backgrounds. Second, the app was designed for use with the iPhone, the CareEvolution team has extended the app for Android users who are currently being enrolled in the study. Third, we restricted our outcomes to survey completion and adherence to mobile devices at 3-month intervals for our current analyses. We plan to examine long-term adherence rates at 6 and 12 months as the study proceeds.

### Conclusions

In this paper, we present our ongoing work to develop a new e-cohort derived from the FHS participants, as well as characteristics of the study population. We report acceptable rates of initial enrollment, high rates of home digital survey completion using a novel eFHS smartphone app, high rates of home HR monitoring using a smartwatch, and modest rates of adherence to weekly BP measurement using a digital BP cuff. Our work and initial findings provide data and show that it is feasible to embed an e-cohort within an ongoing traditional epidemiology study and that middle-aged participants are capable of completing home-based digital surveys and cardiovascular phenotyping using digital devices in the short term. Our initial experience is promising, and we look forward to linking new digital cardiovascular phenotypic data, including daily activity, sedentary time, average HR, and home BP collected in eFHS to validated cardiovascular risk factors and incident cardiovascular outcomes collected in the FHS.

## Appendix

Multimedia Appendix 1 Screenshots of the Welcome, Registration, Survey, and Task Completion screens.

Multimedia Appendix 2 Electronic Framingham Heart Study baseline and follow-up survey components.

Multimedia Appendix 3 Scripted notifications, condition triggers, and notification content deployed through the electronic Framingham Heart Study smartphone application. eFHS: electronic Framingham Heart Study; FHS: Framingham Heart Study.

Multimedia Appendix 4 Characteristics of participants in electronic Framingham Heart Study vs those ineligible or not consenting.

Multimedia Appendix 5 Number and proportion of eligible electronic Framingham Heart Study participants completing all, some, or none of the baseline and 3-month surveys.

Multimedia Appendix 6 Proportion of eFHS participants uploading at least A) one digital Blood Pressure (BP) weekly or B) one heart rate (HR) weekly over the 12 weeks following enrollment, stratified by prevalent hypertension (HTN) and cardiovascular disease (CVD).

## Abbreviations

BP: blood pressure
CES-D: Center for Epidemiologic Studies Depression Scale
e-cohort: electronic cohort
eFHS: electronic Framingham Heart Study
FHS: Framingham Heart Study
HR: heart rate
mHealth: mobile health
NOS: New Offspring Spouse Cohort
OS: operating system
