# Supplementary figures and images for: Design and Preliminary Findings From a New Electronic Cohort Embedded in the Framingham Heart Study
Source: J Med Internet Res. 2019 Mar 1;21(3):e12143. doi: 10.2196/12143 (PMC6418484; doi:10.2196/12143)

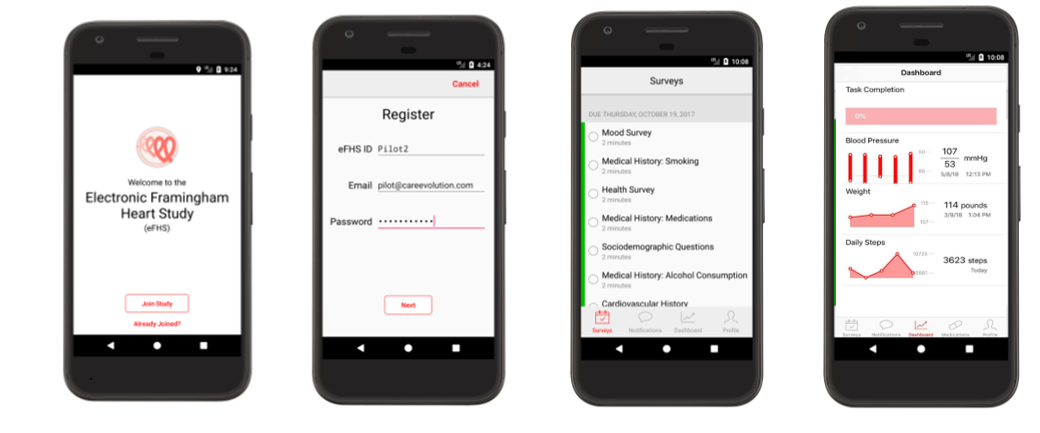

Supplement: Multimedia Appendix 1 [file jmir_v21i3e12143_app1.PNG]

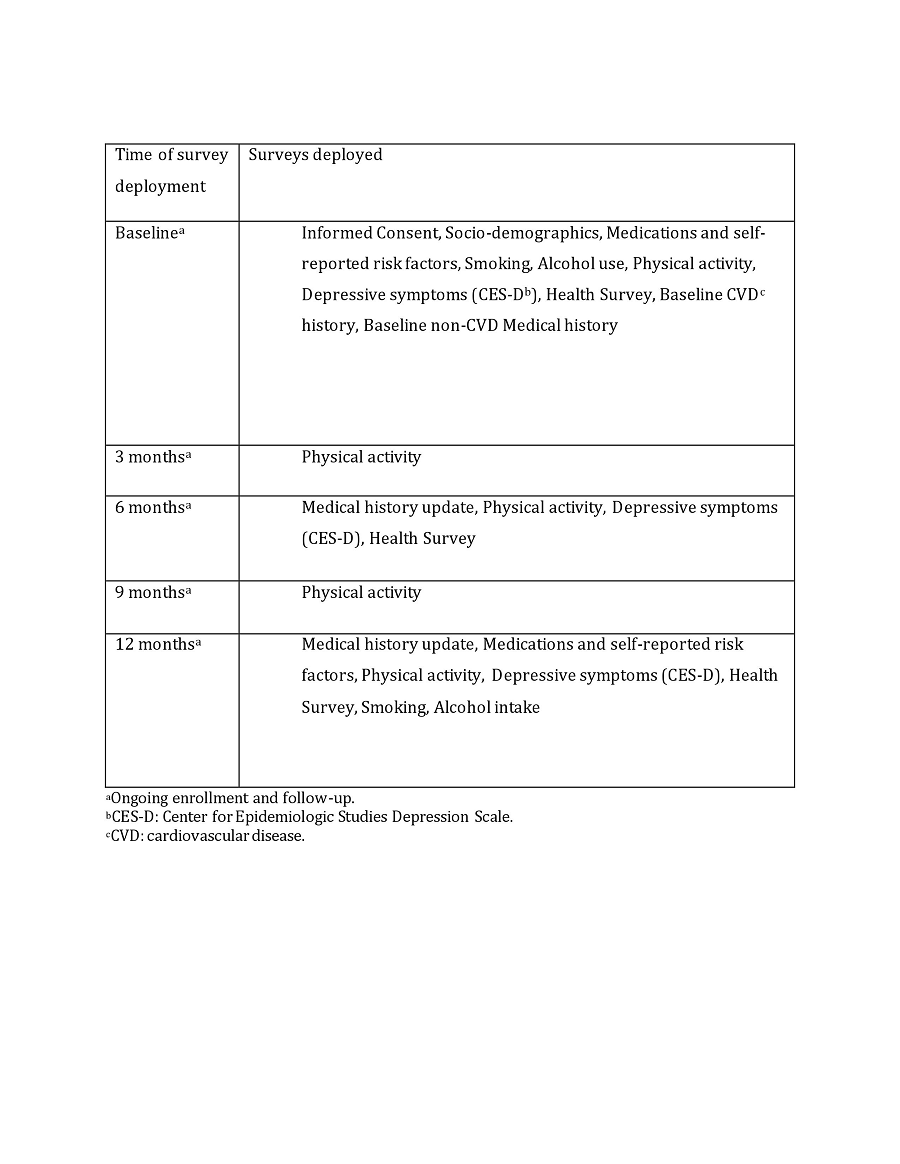

Supplement: Multimedia Appendix 2 [file jmir_v21i3e12143_app2.png]

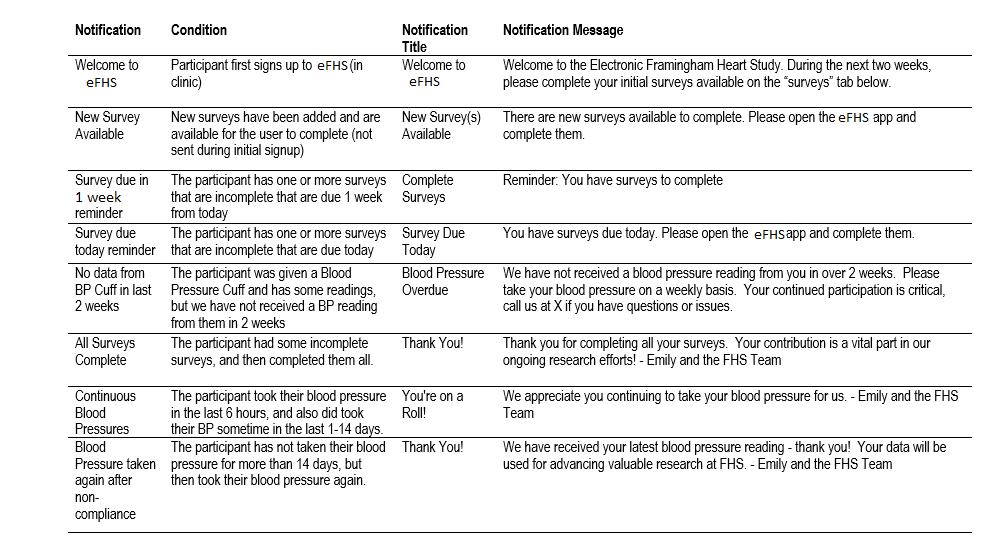

Supplement: Multimedia Appendix 3 [file jmir_v21i3e12143_app3.PNG]

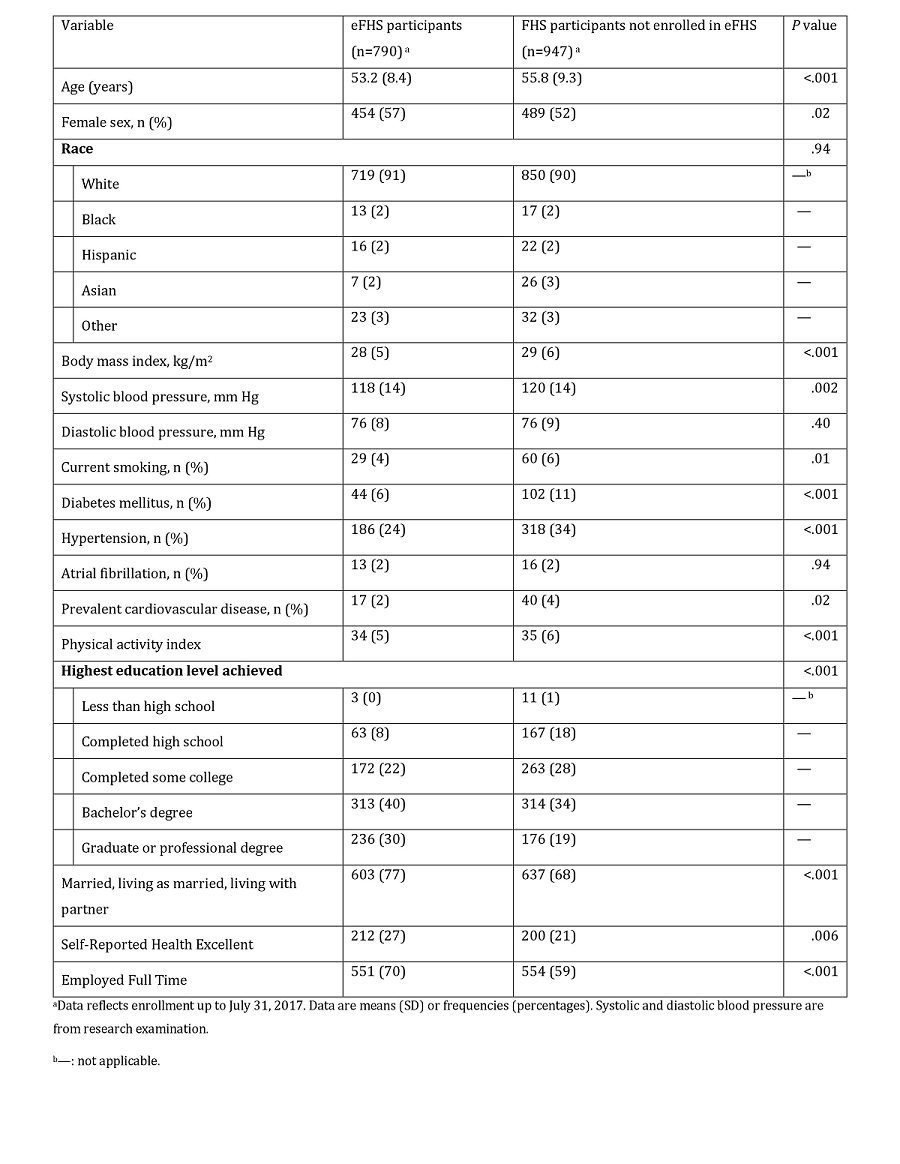

Supplement: Multimedia Appendix 4 [file jmir_v21i3e12143_app4.PNG]

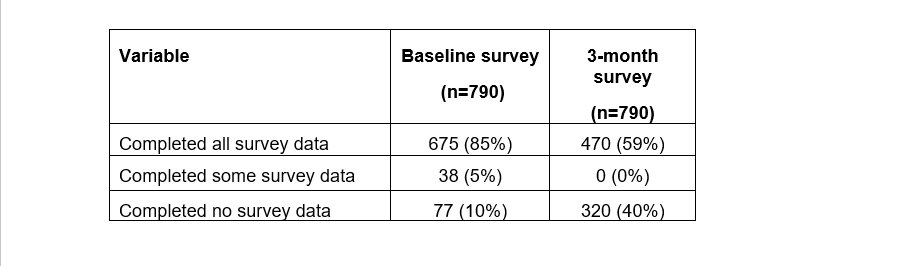

Supplement: Multimedia Appendix 5 [file jmir_v21i3e12143_app5.PNG]

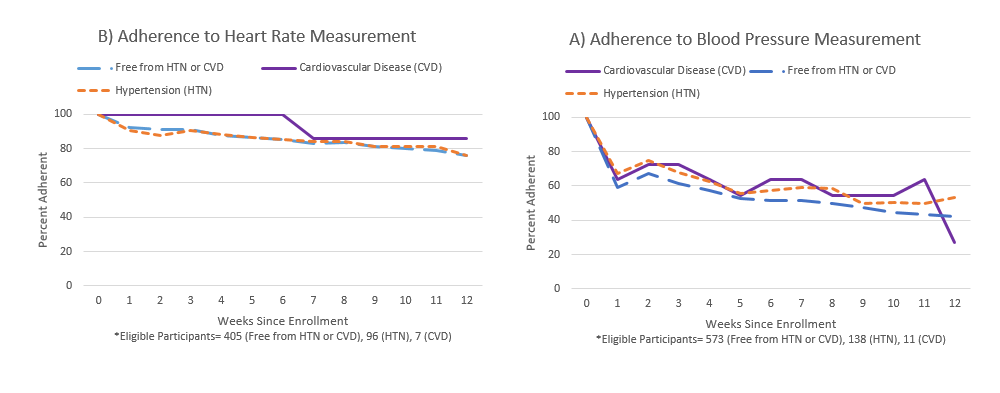

Supplement: Multimedia Appendix 6 [file jmir_v21i3e12143_app6.PNG]
